# High Central Venous Pressure and Right Ventricle Size Are Related to Non-decreased Left Ventricle Stroke Volume After Negative Fluid Balance in Critically Ill Patients: A Single Prospective Observational Study

**DOI:** 10.3389/fmed.2021.715099

**Published:** 2021-08-31

**Authors:** Zhao Hua, Ding Xin, Wang Xiaoting, Liu Dawei

**Affiliations:** Department of Critical Care Medicine, Peking Union Medical College Hospital, Peking Union Medical College, Chinese Academy of Medical Sciences, Beijing, China

**Keywords:** central venous pressure, fluid management, echocardiography, right ventricle size, right ventricle filling

## Abstract

**Background:** Optimal adjustment of cardiac preload is essential for improving left ventricle stroke volume (LVSV) and tissue perfusion. Changes in LVSV caused by central venous pressure (CVP) are the most important concerns in the treatment of critically ill patients.

**Objectives:** This study aimed to clarify the changes in LVSV after negative fluid balance in patients with elevated CVP, and to elucidate the relationship between the parameters of right ventricle (RV) filling state and LVSV changes.

**Methods:** This prospective cohort study included patients with high central venous pressure (CVP) (≥8 mmHg) within 24 h of ICU admission in the Critical Medicine Department of Peking Union Medical College Hospital. Patients were classified into two groups based on the LVSV changes after negative fluid balance. The cutoff value was 10%. The hemodynamic and echo parameters of the two groups were recorded at baseline and after negative fluid balance.

**Results:** A total of 71 patients included in this study. Forty in VI Group (LVOT VTI increased ≥10%) and 31 in VNI Group (LVOT VTI increased <10%). Of all patients, 56.3% showed increased LVSV after negative fluid balance. In terms of hemodynamic parameters at T0, patients in VI Group had a higher CVP (*p* < 0.001) and P(v-a)CO_2_ (*p* < 0.001) and lower ScVO_2_ (*p* < 0.001) relative to VNI Group, regarding the echo parameters at T0, the RV_D_/LV_D_ ratio (*p* < 0.001), DIVC _end−expiratory_ (*p* < 0.001), and ΔLVOT VTI (*p* < 0.001) were higher, while T0 LVOT VTI (*p* < 0.001) was lower, in VI Group patients. The multifactor logistic regression analysis suggested that a high CVP and RV_D_/LV_D_ ratio ≥0.6 were significant associated with LVSV increase after negative fluid balance in critically patients. The AUC of CVP was 0.894. A CVP >10.5 mmHg provided a sensitivity of 87.5% and a specificity of 77.4%. The AUC of CVP combined with the RV_D_/LV_D_ ratio ≥0.6 was 0.926, which provided a sensitivity of 92.6% and a specificity of 80.4%.

**Conclusion:** High CVP and RV_D_/LV_D_ ratio ≥0.6 were significant associated with RV stressed in critically patients. Negative fluid balance will not always lead to a decrease, even an increase, in LVSV in these patients.

## Introduction

In the management of hemodynamic instability, optimal adjustment of cardiac preload is essential for improving stroke volume (SV) and tissue perfusion. Fluid management in critical patients is crucial for prognosis, as both inadequate fluid or fluid overload can lead to negative outcomes (1). In particular, fluid overload and high CVP are associated with poor outcomes in critically ill patients (2). Some studies have concluded that elevated CVP is associated with increased mortality in critically ill patients (3). Conversely, early reductions in CVP during treatment may help maintain good organ function and result in a higher survival rate (4). Negative fluid balance is the most common clinical intervention to reduce CVP. Based on the Frank-Starling mechanism and venous return theory proposed by Guyton, venous return should match cardiac output (CO) as determined by the mean systemic filling pressure (MSFP) and the CVP gradient (5, 6). Changes in CO due to CVP are important concerns for the treatment of critically ill patients. The trend of changes between CVP and CO is not consistent in different conditions. Nevertheless, increases in CO with decreases in CVP occur in routine clinical work, which are indicative of improvement of heart function and pulmonary circulation, especially right heart function.

Right heart function is essential for venous return (7, 8). The healthy human RV fills at or below its unstressed volume, such that RV end-diastolic volume changes occur without any changes in RV diastolic wall stretch. With increased volume loading of the RV, right ventricular end-diastolic pressure (RVEDP) and LVSV both increased. When RV reaches the flat part of the pressure-volume curve, the RV further increases in size will lead a leftward ventricular septal (VS) shift (9). VS shift can result in decreased LVSV, leading to a phenomenon colloquially termed “falling off the Starling curve” (7).

According to the understanding of fluid responsiveness (FR), assessing the filling state of the RV is key to judging the volume status. However, evaluation of the filling state remains a challenge. Dynamic monitoring of CVP and assessment of RV size via echocardiography are currently used as indices of RV filling state (10–13). In our previous retrospective study, the patients with increased cardiac output (CO) and decreased CVP achieved negative fluid balance, but the effect of cardiotonic drugs couldn't be rule out (14). The changes of LVSV after negative fluid balance is still unknown in patients with high CVP. CVP has been used as a surrogate of right ventricle filling pressure, but CVP as a single measure is highly questionable: (1) It remains unclear what level of CVP is deleterious and may be considered a trigger for intervention; (2) The effect of varying intrathoracic pressure in mechanically ventilated patients, and might not reflect preload directly. Several studies have reported that fluid overload can increase RV size [the right to left ventricular end-diastolic dimensions (RV_D_/LV_D_) ratio is ≥0.6] (15–17). Furthermore, the relationship between hemodynamic parameters and LVSV changes after negative fluid balance is unclear. We therefore aimed to assess the changes of LVSV after negative fluid management and to elucidate the relationship between the parameters of right ventricle (RV) filling state and LVSV changes.

## Materials and Methods

### Study Design and Patient Enrollment

This is a *post-hoc* analysis of data collected during a prospective cohort study at the Critical Care Department of Peking Union Medical College Hospital. All patients with abnormally high CVP (i.e., outside the normal range of 0–7 mmHg) within 24 h of ICU admission from July 2017 to December 2017 were included in the study. All the patients authorized us to use their clinical data. The research protocol was reviewed and approved by Ethics Committee of Peking Union Medical College Hospital (PUMCH-S617).

### Inclusion and Exclusion Criteria

The inclusion criteria were (1) CVP ≥8 mmHg and (2) age >18 years. The exclusion criteria were (1) negative balance therapy was not performed; (2) non-curative goals of therapy, (3) a history of cardiac disease, pulmonary hypertension, or precaval malformations, and (4) abdominal hypertension.

### Study Protocol

All patients were treated as follows:

All enrolled patients underwent the routine procedures of the Critical Care Department of Peking Union Medical College Hospital. Arterial and venous lines were inserted. Time 0 (T0) is defined as the time of patient enrollment, and Time 1 (T1) is defined as a negative fluid balance of 500 ml. Central venous and arterial blood gases analysis were performed at T0 and T1. Patients were divided into two groups according to the LVOT VTI changes after negative fluid balance: VI Group comprised patients with ΔLVOT VTI(T1-T0)/T0 LVOT VTI ≥10% (VI) and VNI Group comprised patients with ΔLVOT VTI(T1-T0) /T0 LVOT VTI <10%(VNI).

**Figure 1:**
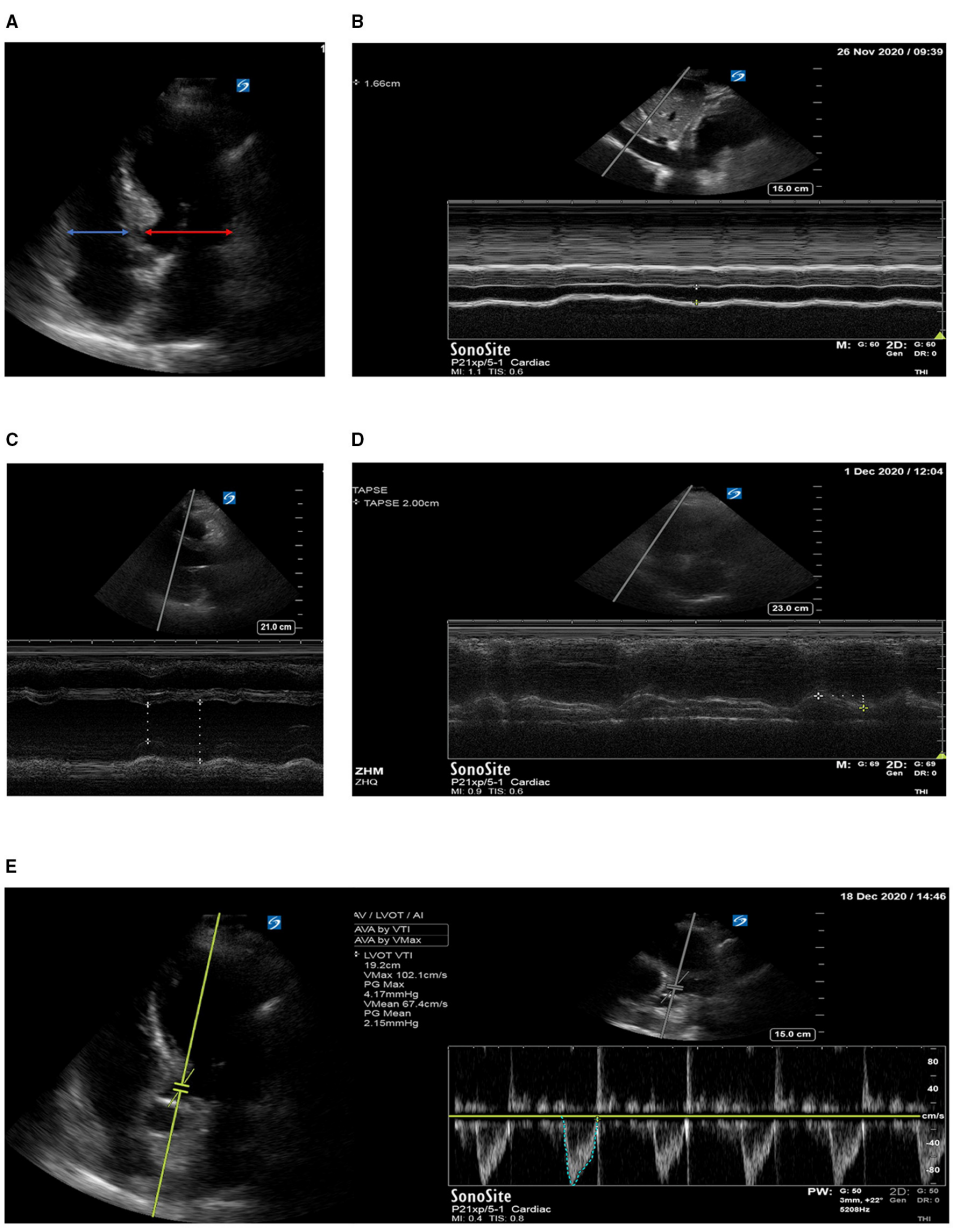
**(A)** Right to left ventricular diastolic dimensions ratio (RV_D_/LV_D_ ratio); **(B)** diameter of the inferior vena cava; **(C)** left ventricular eject fraction EF; **(D)** tricuspid annular plane systolic excursion TPASE; **(E)** left ventricular outflow tract velocity time integral VTI.

- *Hemodynamic monitoring methods*: The lines of central venous and arterial were inserted, CVP, central venous oxygen saturation (ScVO_2_), central venous-arterial carbon dioxide difference [P(v-a)CO_2_], and serum lactate levels (lac) were tested (detailed were showed in Supplementary Material 1). Bladder pressure was used as a surrogate of intra-abdominal pressure (IAP), IAP ≥12 mmHg is defined as abdominal hypertension.
- *Echocardiography*: Echocardiography was also performed at T0 and T1 by competent attending physicians or fellows, who had at least 3 years of experience in echocardiography performance and interpretation. An ultrasound system equipped with an array probe (X-Porte, Sonosite, Bothell, WA, USA) was used. At least five standard views (acoustic windows) were obtained and recorded for each scan (Figure 1) (detailed were showed in Supplementary Material 1). All the reviews were confirmed by two competent attending physicians.
- *Clinical treatment*: The method of negative fluid balance (application of diuretic drugs or continuous renal replacement therapy) was determined by the physician. No changes were made that may cause changes in CVP. In the case of patient hypoxia, the inhaled oxygen concentration was adjusted to ensure SPO_2_ >95%, PaO_2_ >60 mmHg.

### Data Collection

Clinical data were extracted from the ICU computerized database and medical records, including patients' socio-demographic data, biometric parameters, comorbidities, respiratory support mode, and Acute Physiology and Chronic Health Evaluation II score, SOFA score. Hemodynamic parameters [heart rate, mean arterial blood pressure, CVP, ScVO_2_, P(v-a)CO_2_, and lac] and echo parameters [such as tricuspid annular plane systolic excursion (TAPSE), diameter of the inferior vena cava (DIVC _end−expiratory_), left ventricular eject fraction (LVEF), RV_D_/LV_D_ ratio, left ventricle outflow tract velocity-time integral (LVOT VTI), mitral annular plane systolic excursion (MAPSE), mitral diastolic filling Eatly (Em), mitral diastolic filling Atrial (A m), and early diastolic velocity of the mitral annulus Ea m] were recorded at T0 and T1, were also recorded at the same time. The primary outcome of the study was to clarify LVOT VTI changes after negative fluid management in patients with high CVP. The secondary outcome was to evaluate the relationship between the relationship between the parameters of RV filling state and LVOT VTI changes after negative fluid management.

### Statistical Analysis

Statistical analysis was performed using SPSS software version 20.0 for Windows (IBM, Armonk, NY). Considering this is a *post-hoc* analysis of data, sample size was calculated through events per variable (EPV) method. EPV of 5–10 is usually used to estimate sample size in the literature. At most 6 candidate variables could be included in the multivariate modeling process when half of patients will present positive result. The data distribution test and the homogeneity of variance test were performed on the data. Results for continuous variables with a normal distribution (e.g., age, Acute Physiology and Chronic Health Evaluation II score) are reported as the mean ± standard deviation. Student's *t*-test was used to compare means between two groups. Results for continuous variables that were not normally distributed are reported as the median (25th and 75th percentiles) and compared using non-parametric tests. Qualitative data were expressed as proportions; testing for differences was performed using a chi-square test or Fisher's exact test. The paired sample *t*-test was used for comparisons between groups before and after treatment. The correlation between RV variables and LVOT VTI changes was analyzed using Pearson correlation analysis. Risk factors associated with VI were identified in univariate and multivariate logistic regression analysis, variables with a *P* < 0.2 were subjected to a multivariate analysis with backward stepwise models to measure the odd risk (OR) and 95% confidence intervals (CIs). Receiver operating characteristic (ROC) curves were used to determine the ability of the indices to predict LVOT VTI increase >10% after negative fluid balance. The areas under the ROC curves (AUCs) were compared using DeLong's test. The AUC, sensitivity, and specificity are expressed as values with 95% CI. A *p* < 0.05 was considered to be statistically significant.

## Results

### Demographic and Clinical Characteristics of Patients

**Figure 2:**
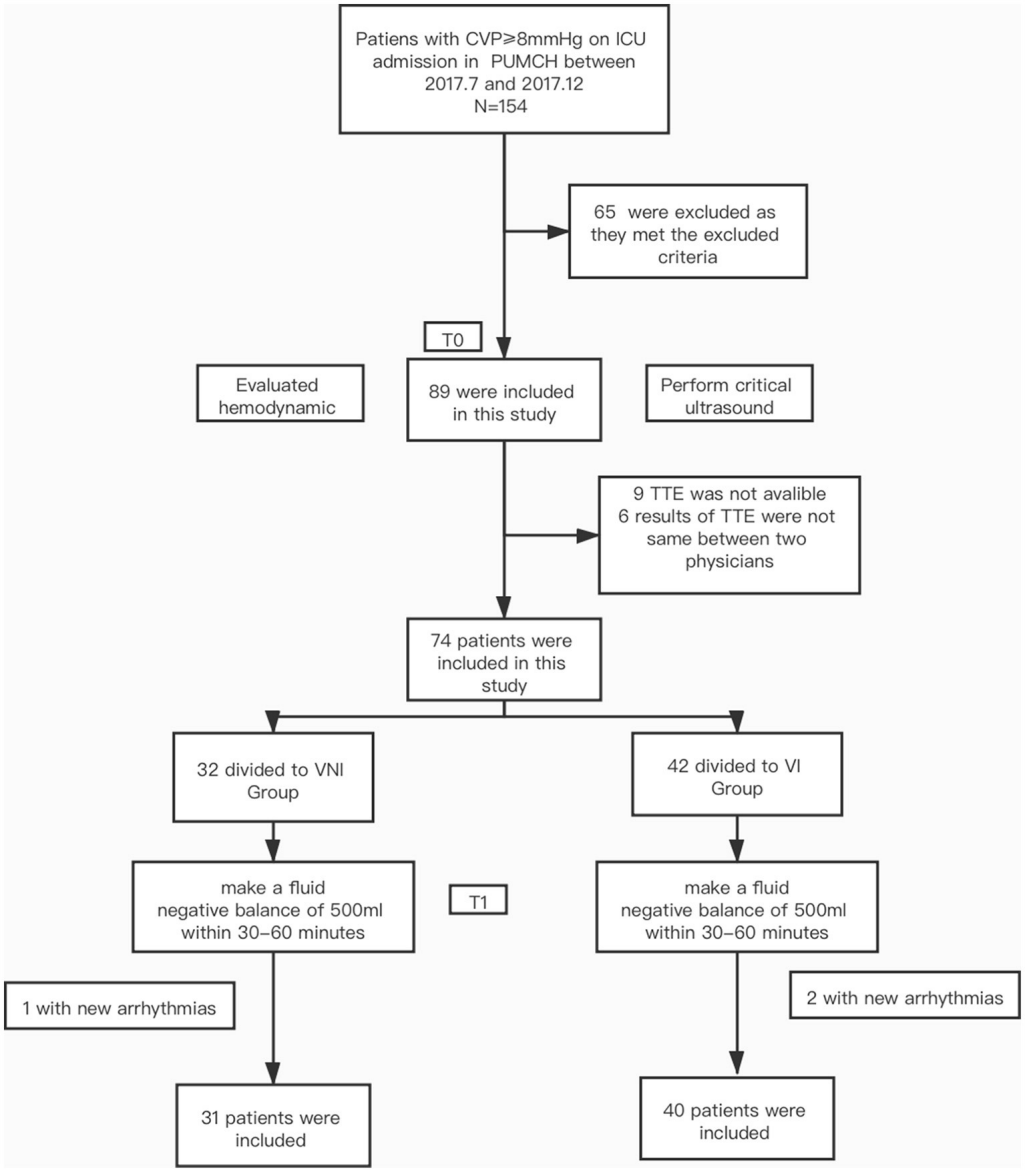
Flow chart.

During the study period, a total of 154 patients were admitted with CVP ≥8 mmHg. Of these, 65 did not meet the study criteria (28 didn't undergo negative fluid management, 26 had underlying heart disease, 5 had abdominal hypertension, 6 with non-curative goals of therapy). In addition, 9 were excluded due to poor TT image quality or incomplete image acquisition; 6 were excluded due to inconsistent judgments of the ultrasound results by the two physicians; and 3 were excluded due to new tachyarrhythmia during the trial. Thus, the final sample for analysis comprised 71 patients (33 males, 38 females) (Figure 2). Overall, 40 (56.3%) patients were grouped to VI Group, 31 (43.7%) patients were grouped to VNI Group. The demographical and clinical characteristics of all patients are shown in Table 1. Except for the P/F ratio (*p* < 0.05), there were no significant differences in other demographic characteristics between the two groups. In terms of hemodynamic parameters at T0, patients in VI Group had a higher CVP and P(v-a) CO_2_ and lower ScVO_2_ relative to VNI Group (all *p* < 0.05). No group differences were observed for HR, MAP, or lactate levels. Regarding the echo parameters at T0, the RV_D_/LV_D_ ratio ≥0.6, DIVC _end−expiratory_, ΔLVOT VTI were higher, while T0 LVOT VTI was lower, in VI Group (all *p* < 0.05). There were no group differences in LV systolic and diastolic function, RV systolic fuction, and tricuspid regurgitation as shown in Table 2.

**Table 1:** The demographic and clinical characteristics of the patients included in this study at T0.

**Variable**	**All patients (*N* = 71)**	**VI group (*N* = 40)**	**VNI group (*N* = 31)**	***P-*value**
Gender (male/female)	71 (33/38)	40 (18/22)	31 (15/16)	0.321
Age (years)	49 ± 16	48 ± 16	50 ± 14	0.334
SOFA Score (mean ± SD)	9.1 ± 4.5	9.2 ± 4.5	8.9 ± 4.4	0.384
APACHE II Score (mean ± SD)	16 ± 5	17 ± 5	15 ± 5	0.173
**Underlying disease** ***n*** **(%)**
Coronary artery disease	13 (18.3)	7 (17.5)	6 (19.4)	0.540
Hypertension	28 (39.4)	17 (42.5)	11 (35.5)	0.362
Diabetes mellitus	24 (33.8)	16 (40)	8 (25.8)	0.158
Choric renal failure	12 (16.9)	8 (20)	4 (12.9)	0.218
Choric liver failure	4 (5.6)	3 (7.5)	1 (3.2)	0.409
Stroke	8 (11.3)	4 (10)	4 (12.9)	0.493
Cancer	6 (8.5)	4 (10)	2 (6.5)	0.466
**Primary disease** ***n*** **(%)**
Sepsis	34 (47.9)	20 (50)	14 (45.1)	0.435
gastrointestinal bleeding	3 (4.2)	2 (5)	1 (3.2)	0.596
Traumatic brain injuries	3 (4.2)	2 (5)	1 (3.2)	0.596
Acute coronary syndrome	9 (12.7)	5 (12.5)	4 (12.9)	0.616
Post-operative of gastrointestinal tumors	5 (7.0)	3 (7.5)	2 (6.5)	0.621
Post-operative of urinary system	10 (14.1)	4 (10)	6 (19.4)	0.217
Post-operative of gynecologic cancer	7 (9.9)	4 (10)	3 (9.7)	0.642
RASS Score	−2.33 ± 1.16	−2.45 ± 1.23	−2.37 ± 1.19	0.674
**Proportion of organ failure**
PaO_2_/FiO_2_ (mmHg)	240.62 ± 46.19	214.10 ± 39.63	274.84 ± 28.09	<0.001
Acute kidney injury *n* (%)	16 (22.5)	9 (22.5)	7 (22.5)	0.607
Acute myocardial injury *n* (%)	10 (14.1)	6 (15)	4 (12.9)	0.541
Acute liver injury *n* (%)	4 (5.6)	3 (7.5)	1 (3.2)	0.409
**Interventions**
CRRT (*n* %)	8 (11.3)	5 (12.5)	3 (9.7)	0.507
**Ventilation modes** ***n*** **(%)**
Non-invasive ventilation	13 (18.3)	8 (20)	5 (23.8)	0.256
Invasive ventilation	58 (81.7)	32 (80)	26 (74.2)	0.354
Vasoactive drugs (*n* %)	36 (50.7)	20 (50.0)	16 (51.6)	0.542
Fluid input (T0–T1) (ml)	86 ± 15	85 ± 14	87 ± 16	0.482
T1–T0 time (minute)	63 ± 14	58 ± 15	64 ± 16	0.275

*Quantitative data are expressed as the mean ± SD or median interquartile (25–75) %. Qualitative data are expressed as n (%). APACHE II, Acute Physiology and Chronic Health Evaluation II; Sofa Score, Sequential Organ Failure Assessment Score; PaO_2_, arterial oxygen pressure; FiO_2_, fraction of inspiration oxygen; CRRT, continue renal replacement therapy; RASS Score, Richmond Agitation-Sedation Scale Score*.

**Table 2:** The hemodynamic and echo characteristics of all the included patients at T0.

**Variable**	**All patients (*N* = 71)**	**VI group (*N* = 40)**	**VNI group (*N* = 31)**	***P-*value**
**Hemodynamic parameters**
Central venous pressure CVP (mmHg)	11.42 ± 2.27	12.74 ± 1.98	9.74 ± 1.32	<0.001
Mean arterial blood pressure MAP (mmHg)	75.70 ± 8.724	75.72 ± 8.48	75.68 ± 8.06	0.981
Heart rate HR (bpm)	94.06 ± 15.86	95.83 ± 16.28	91.77 ± 15.24	0.289
P(v-a)CO_2_ (mmHg)	5.42 ± 1.98	6.43 ± 1.63	4.11 ± 1.59	<0.001
ScVO_2_ %	68.44 ± 7.71	64.32 ± 5.45	73.76 ± 6.94	<0.001
Arterial blood lactate level lac (mmol/l)	3.23 ± 0.79	3.30 ± 0.83	3.13 ± 0.73	0.353
**Echo parameters**
LVOT VTI (T0)	18.38 ± 2.72	16.94 ± 1.59	20.23 ± 2.76	<0.001
Left ventricular eject fraction EF %	62.08 ± 5.04	62.13 ± 4.99	62.03 ± 5.17	0.939
MAPSE (cm)	1.34 ± 0.28	1.33 ± 0.21	1.36 ± 0.37	0.428
E/A m	1.12 ± 0.36	1.21 ± 0.57	1.08 ± 0.29	0.539
E m (cm/s)	68 ± 19	71 ± 19	65 ± 11	0.029
A m (cm/s)	62 ± 20	63 ± 22	61 ± 18	0.673
E/Ea m	10.52 ± 2.19	11.74 ± 1.36	10.09 ± 3.27	0.126
Tricuspid vale regurgitation (TVR) *n* (%)	29 (40.4)	19 (47.5)	10 (32.3)	0.167
+^*^n (%)	26 (36.6)	17 (42.5)	9 (29.0)	0.094
++*^*^n (%)	3 (4.2)	2 (5)	1 (3.2)	0.243
DIVC _end−expiratory_ (cm)	1.93 ± 0.28	2.08 ± 0.25	1.75 ± 0.21	<0.001
TAPSE (cm)	2.16 ± 0.26	2.15 ± 0.28	2.18 ± 0.25	0.765
RV_D_/LV_D_ ratio ≥0.6 *n* (%)	35 (49.3)	29 (72.5)	6 (20)	<0.001

*P(v-a)CO_2_, central venous-arterial carbon dioxide difference; ScVO_2_, central venous oxygen saturation; MAPSE, mitral annular plane systolic excursion; DIVC _end−expiratory_, diameter of the inferior vena cava; TPASE, tricuspid annular plane systolic excursion; LVOT VTI, T0 left ventricle outflow tract velocity time integral T0; RV_D_/LV_**D**_ ratio, right to left ventricular end-diastolic dimensions ratio; Em, mitral diastolic filling Eatly; A m, mitral diastolic filling Atrial; Ea m, early diastolic velocity of the mitral annulus*.

* *TVR <2.8 m/s*. *^*^TVR ≥2.8 m/s.

### Comparison of Hemodynamic Parameters at T0 and T1 Between Patients With Rv Dilation or Not

The analysis showed there was a significant difference in proportion of RV_D_/LV_D_ ratio ≥0.6 (RV dilation) between the two groups. We compared the hemodynamic parameters at T0 and T1 between patients with RV_D_/LV_D_ ratio ≥0.6 (RV dilation) or not (non-RV dilation).

**Figure 3:**
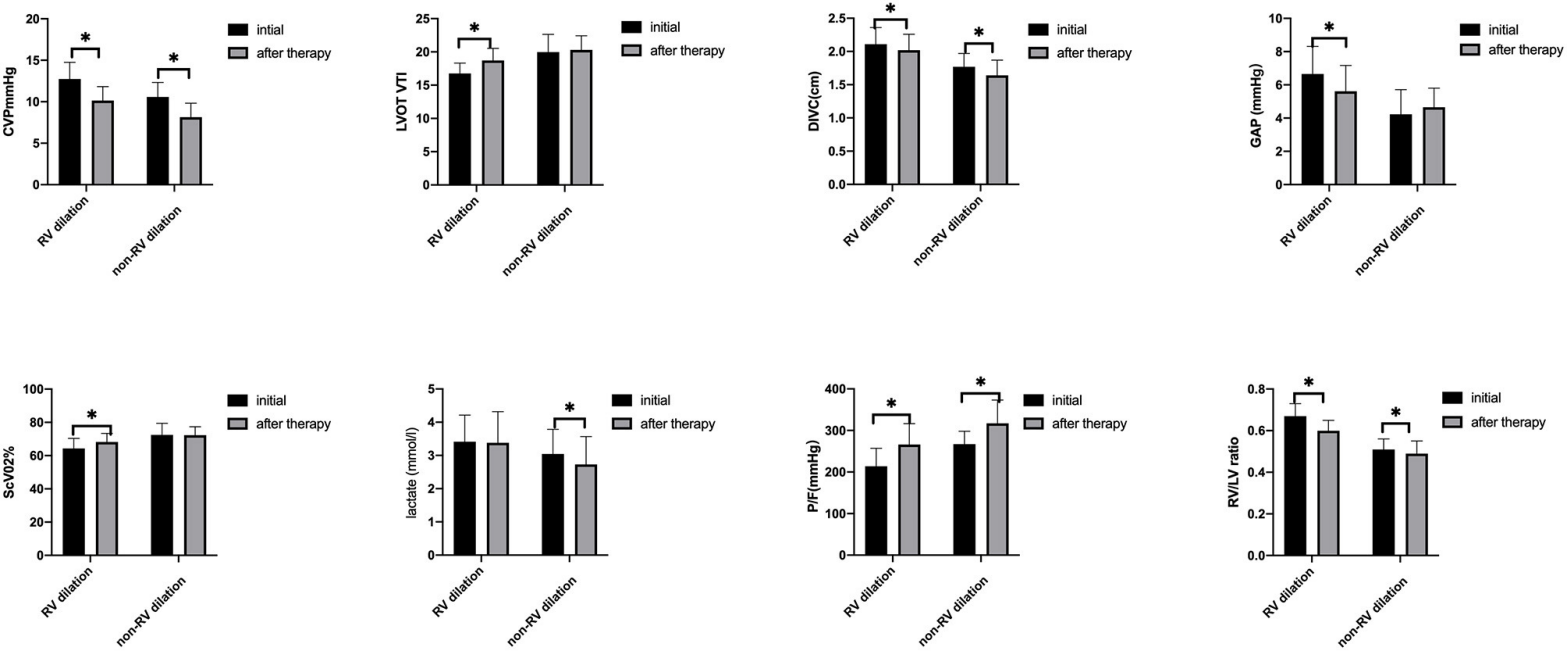
Hemodynamic parameters between T0 and T1 in the two groups, *indicates a *p* < 0.05 between the two groups. CVP, central venous pressure; LVOT VTI, left ventricular outflow tract velocity time integral; DIVC _end−expiratory_, diameter of the inferior vena cava; P(V-A)CO_2_, central venous-arterial carbon dioxide difference; ScVO2, central venous oxygen saturation; P/F, arterial partial pressure of oxygen to fractional concentration of inspired oxygen; RV_D_/LV_D_ ratio, right to left ventricular diastolic dimensions ratio.

As shown in Figure 3, CVP, DIVC _end−expiratory_, and RV_D_/LV_D_ ratio decreased significantly in both groups after negative fluid management (*p* < 0.05). None of the patients in our study experienced tissue perfusion insufficiency. Flow related parameters [LVOT VTI, P(v-a)CO_2_, ScVO_2_] improved in patients with RV_D_/LV_D_ ratio ≥0.6 (*p* < 0.05), and the lactate level decreased in patients with RV_D_/LV_D_ ratio <0.6 (*p* < 0.05). In addition, the P/F ratio increased significantly in both groups (*p* < 0.05).

### Correlation Between RV Parameters and Δ LVOT VTI/T0 LVOT VTI

**Figure 4:**
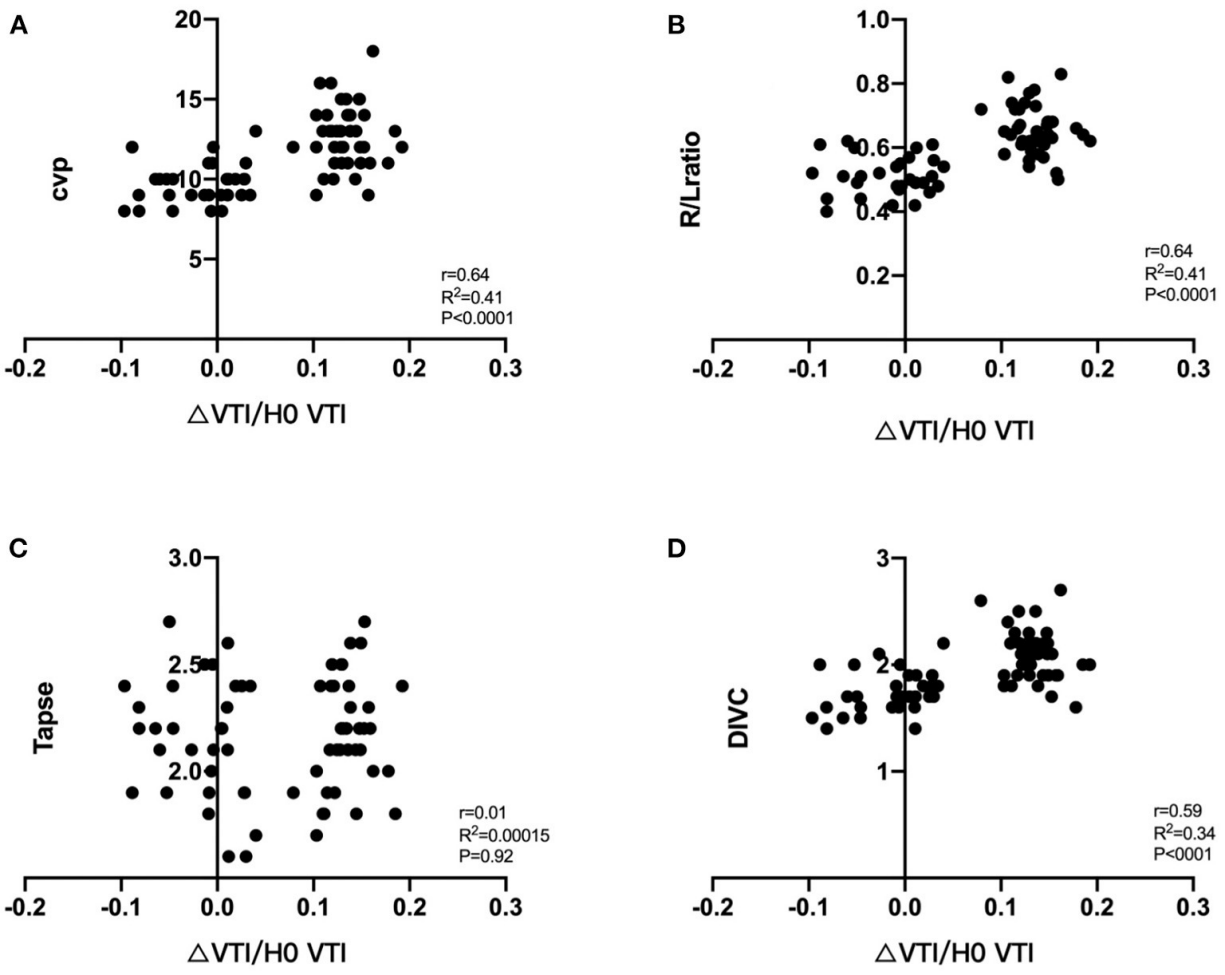
Correlation between ΔLVOT VTI/T0 LVOT VTI and RV parameters, ΔLVOT VTI/T0LVOT VTI = T1LVOT VTI-T0 LVOT VTI/T0LVOT VTI, its relationship with CVP **(A)**, RV_D_/LV_D_ ratio **(B)**, TAPSE **(C)**, DIVC _end−expiratory_ **(D)**. VTI, velocity time integral; CVP, central venous pressure; TPASE, tricuspid annular plane systolic excursion; RV_D_/LV_D_ ratio, right to left ventricular diastolic dimensions ratio; DIVC _end−expiratory_, diameter of the inferior vena cava.

Figure 4 presents the individual parameter values for RV and Δ LVOT VTI/T0 LVOT VTI among all patients. CVP, RV_D_/LV_D_ ratio, and DIVC _end−expiratory_ were significantly correlated with Δ LVOT VTI/T0 LVOT VTI [*r* = 0.64 (*p* < 0.05), 0.64 (*p* < 0.053), and 0.59 (*p* < 0.05), respectively]. By contrast, no relationship was observed between LV systolic and diastolic functions, RV systolic function and tricuspid regurgitation and Δ LVOT VTI/T0 LVOT VTI.

### Risk Factors for a LVOT VTI Increase ≥10% at T1

The multifactor logistic regression analysis was used to examine possible risk factors for the changes of LVOT VTI after negative fluid management. All relevant variables were taken into account (including demographics and clinical characteristics, hemodynamic and ECHO findings). P(v-a) CO_2_ and ScVO_2_ were excluded, as they are derived variables of LVOT VTI, P/F was exclude as it was consequence variables of RV stressed. The results of univariate analysis suggested that the *P-*value of CVP, RV_D_/LV_D_ ratio, LVOT VTI, DIVC _end−expiratory_ and E m was <0.2 (Table 3). Then these variables were subjected to the multivariate logistic regression analysis with backward stepwise model. The results suggested that a high CVP and RV_D_/LV_D_ ratio were significant associated with LVSV increase after negative fluid balance in critically patients (*p* < 0.05). The OR of CVP and RV_D_/LV_D_ ratio were 2.425 (95% CI, 1.458–4.003) and 8.588 (95% CI, 1.947–37.887), respectively (Table 3).

**Table 3:** Logistic regression analysis for possible risk factors of LVOT VTI increased ≥10% after negative fluid balance.

**Variables**	**Univariate logistic regression analysis**				**Univariate logistic regression analysis**			
	**OR**	**95% CI for OR**		***P* value**	**OR**	**95% CI for OR**		***P* value**
		**Lower**	**Upper**			**Lower**	**Upper**	
Central venous pressure CVP (mmHg)	2.920	1.813	4.703	<0.001	2.425	1.458	4.033	0.001
RV_**D**_/LV_**D**_ ratio ≥0.6	23.250	6.427	84.112	<0.001	8.588	1.947	37.887	0.005
DIVC _end−expiratory_ (cm)	860.978	34.924	21225.283	<0.001	1.846	0.012	294.155	0.813
TAPSE (cm)	1.320	0.221	7.902	0.761				
LVOTs VTI T0	0.495	0.351	0.697	<0.001	0.689	0.452	1.051	0.084
LVEF (%)	1.004	0.914	1.102	0.938				
MAPSE (cm)	1.123	0.879	1.105	0.687				
E m (cm/s)	1.049	1.003	1.098	0.036	1.019	0.953	1.090	0.574
Am (cm/s)	1.006	0.846	1.423	0.635				
E/Ea	0.987	0.684	1.256	0.578				
Tricuspid vale regurgitation (TVR)			
+	0.553	0.204	1.500	0.245				
++	0.633	0.055	7.323	0.715				
Mean arterial blood pressure MAP (mmHg)	1.001	0.945	1.060	0.981				
Heart rate HR (bpm)	1.017	0.986	1.049	0.286				
Arterial blood lactate level lac (mmol/l)	1.337	0.728	2.455	0.348				
**Demographics and clinical characteristics**			
SOFA Score	1.012	0.910	1.126	0.821				
APACHE II Score	0.941	0.856	1.034	0.209				
**Primary disease**			
Sepsis/septic shock	0.824	0.322	2.109	0.824				
Acute coronary syndrome	1.037	0.254	4.236	0.960				
Traumatic brain injuries	0.633	0.055	7.323	0.715				
Gastrointestinal bleeding	0.633	0.055	7.323	0.715				

*MAPSE, mitral annular plane systolic excursion; DIVC _end−expiratory_, diameter of the inferior vena cava; TPASE, tricuspid annular plane systolic excursion; LVOT VTI T0, left ventricle outflow tract velocity time integral T0; RV_D_/LV_**D**_ ratio, right to left ventricular end-diastolic dimensions ratio; Em, mitral diastolic filling Eatly; A m, mitral diastolic filling Atrial; Ea m, early diastolic velocity of the mitral annulus; APACHE II, Acute Physiology and Chronic Health Evaluation II; Sofa Score, Sequential Organ Failure Assessment Score*.

### Effect of Risk Parameters for Predicting a LVOT VTI Increase >10% at T1

**Figure 5:**
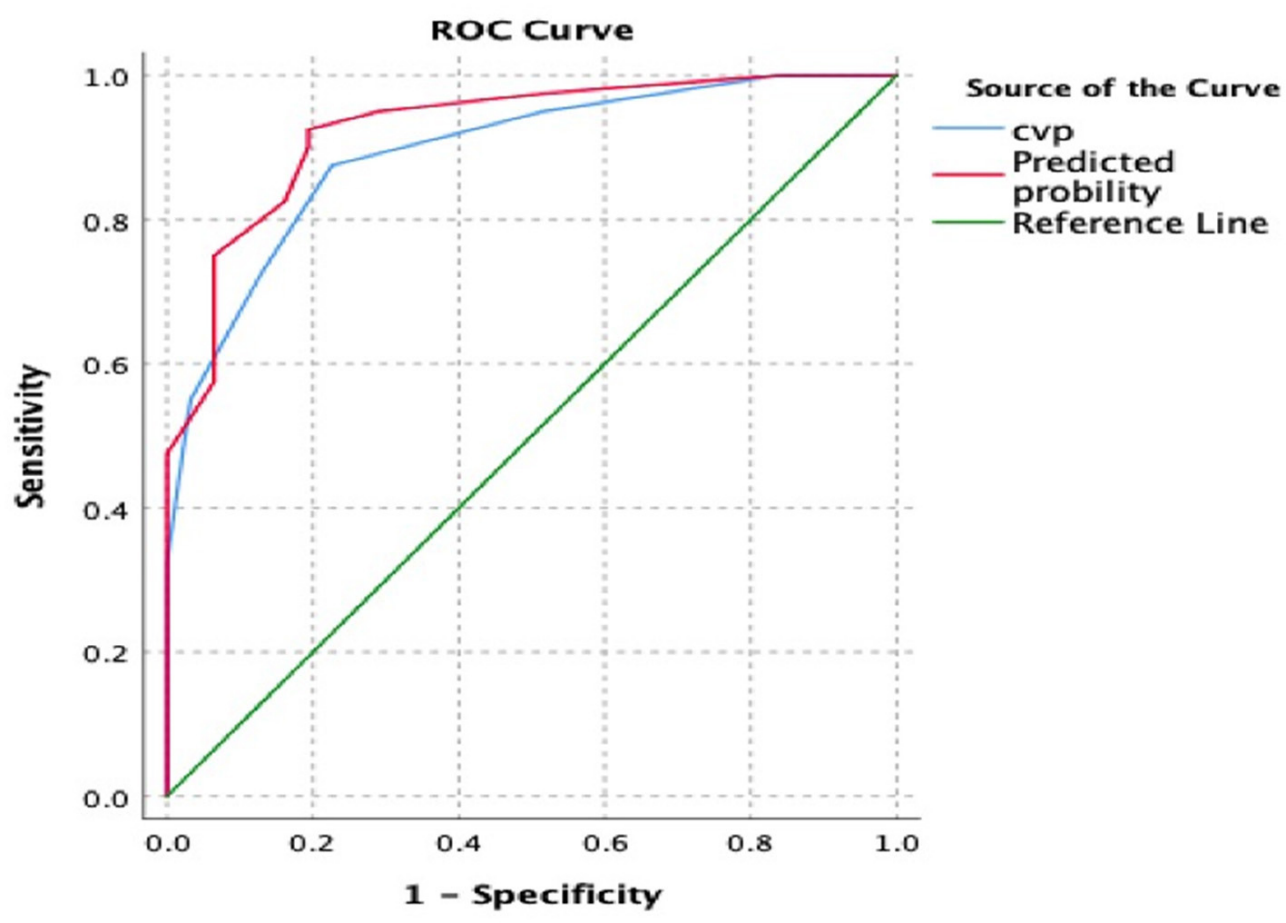
The receiver operating characteristic (ROC) curves and the area under the ROC curve (AUC) for prediction of VTI increase after negative fluid balance. The “red model” included CVP and RV_D_/LV_D_ ratio, while the “blue model” only included CVP. The AUC was 0.926 and 0.894, respectively. VTI, velocity time integral; CVP, central venous pressure; RV_D_/LV_D_ ratio, right to left ventricular diastolic dimensions ratio.

The AUC of CVP for predicting a LVOT VTI increase >10% at T1 was 0.883 (95% CI 0.804–0.902). The best diagnostic threshold was 10.5 mmHg, which provided a sensitivity of 87.5% and a specificity of 77.4% (Figure 5).

The regression equation for all of the risk parameters is:

Logit (P) = −10.474 + CVP ^*^ 0.886 + RV_D_/LV_D_ ratio ^*^ 2.854 (≥0.6 = 1, <0.6 = 0).

The AUC of CVP combined with RV_D_/LV_D_ ratio for predicting a LVOT VTI increase >10% at T1 was 0.926 (95% CI 0.866–0.96). The best diagnostic threshold was 0.3689, which provided a sensitivity of 92.5% and a specificity of 80.6% (Figure 5).

## Discussion

The main findings of our study are as follows: (1) Negative fluid balance will not always lead to a decrease, even an increase, in patients with high CVP, especially combined with RV_D_/LV_D_ ratio ≥0.6. (2) The underling mechanism may be related to the filling state of RV. Our results suggested high CVP and RV_D_/LV_D_ ratio (≥0.6) were significantly associated with RV stressed, CVP ≥10.5 combined with RV_D_/LV_D_ ratio ≥0.6 can predict higher LVSV derived from negative fluid balance. (3) We found that the patients with high CVP can benefit from oxygenation index improvement through dehydration treatment, CVP can be used as a clinical safety mechanism to avoid fluid overload.

In our study, the intervention was performed in a short period of time (63 ± 14 min), while other factors affecting CVP were unchanged. Notably, 56.3% of our patients showed increased VTI after negative fluid balance, 43.7% showed decreased or unchanged VTI, which is not exactly the same as the comment cognition. As the Starling curve does not have descending branches, it cannot explain the increase in LVSV after negative fluid management. Thus, we assume that patients experienced RV stressed. A negative fluid balance can reduce RV volume, resulting in a rightward VS shift, an increase in left ventricular end-diastolic volume, and an increase in LVSV.

In terms of the hemodynamic and echo parameters in our cohort, CVP, P(v-a)CO_2_, ScVO2, RV_D_/LV_D_ ratio, LVOT VTI, and DIVC _end−expiratory_ differed significantly between the two groups at T0. A high CVP value and RV_D_/LV_D_ ratio ≥0.6 were significantly associated with RV stressed. Antoine Vieillard-Baron also found RV failure was frequent (42% of cases) when defined by the association of RV dilatation (RV/LV EDA ≥0.6) with systemic congestion (CVP ≥8 mmHg) in septic shock patients, and only 20–30% patients responded to fluid (18, 19). Due to its geometrical complexity, assessment of RV volume is a very difficult task. Although quantitative validation is lacking, RV_D_/LV_D_ ratio has prognostic value in multiple patient populations, including acute pulmonary embolism, idiopathic pulmonary arterial hypertension, and post-left ventricular assist device implantation, the correlation of RV linear dimensions with RV end-diastolic volumes appears to worsen with increased preload (20–22). RV_D_/LV_D_ ratio has been shown to be an indicator of RV size, and can thus provide reliable information about RV shape and size. A ratio ≥0.6, regardless of whether RV is within the normal reference limits, may relate to certain conditions such as RV stressed (20). Our results are similar to those of previous studies (23). Our measurement method is more clinically operable and repeatable. However, in patients with pulmonary embolism and chronic pulmonary hypertension, only RVEDA/LVEDA >1 indicates RV stressed (24, 25). When R/V ratio is applied clinically, it should be considered in combination with the patient's underlying disease and ventricular septal morphology. We also identified high CVP was significantly associated with RV stressed. The gold standard for evaluating RV filling pressure is invasive monitoring using a centrally placed venous catheter (26). Since the filling pressure and LVSV of the RV do not have a linear relationship, it has recently been acknowledged that CVP is ineffective for evaluating a patient's fluid responsiveness (27–29). While the absolute value of CVP alone cannot predict fluid responsiveness, it is necessary to understand that CVP is a marker of pressure and a regulating factor of venous return. Thus, an increase in CVP can be used as a clinical safety mechanism to avoid fluid overload and high RV filling pressure (30). In the present study, we found that a high CVP may reflect that the RV volume load has exceeded the normal range; failure to appreciate this limit may result in a VS rightward shift and reduced LVSV. It has been proposed that, once CVP has exceeded 10–14 mm Hg in non-intubated patients with acute RV myocardial infarction, further volume loading is detrimental. A mean CVP >14 mmHg is almost always associated with a reduced RVSWI (31, 32). Garcia-Montilla et al. (33) reported that the optimal RV filling pressure in patients with acute respiratory distress syndrome (ARDS) is 13 ± 2 mm Hg. Furthermore, they demonstrated that once CVP reaches 15 mmHg, further increments in filling pressure did not increase RVSPG; rather, due to overstretching of myocardial fibers, RVSPG decreased. These values may be considered the optimal RV filling pressure in patients with acute RV infarction or ARDS. Our results suggest that CVP >10.5 mmHg can predict whether VTI increases after a negative fluid balance in patients without underlying cardiac disease with high sensitivity but low specificity, when combined with RV_D_/LV_D_ ratio ≥0.6 the predictive ability improved.

Notably, none of our patients experienced tissue perfusion insufficiency after negative fluid balance. However, the oxygenation index improved in both groups—especially VI Group. It is well-known that fluid overload may lead to pulmonary edema and failure of weaning from mechanical ventilation. A milestone study by National Heart, Lung, and Blood Institute Acute Respiratory Distress Syndrome (ARDS) Clinical Trials Network et al. (34) showed that a conservative fluid management protocol aimed to lower CVP resulted in a major reduction in net fluid balance, improving lung function and shortening the duration of mechanical ventilation. Clinicians should be alert to high CVP as it may indicate increased RV tension and leftward VS, potentially leading to increased left ventricular filling pressure and pulmonary edema. Accurate fluid therapy for patients with high CVP (systemic congestion) will not lead to hypoperfusion, but will be beneficial for other organs.

This study has some limitations. First, it was a single- center, prospective cohort study. In addition, the sample size is limited, and may have thus systematically excluded some participant groups. As a pragmatic study, this population had similar characteristics to previous clinical audits using the same inclusion criteria. Although RV_D_/LV_D_ ratio entered to the equation and the *P* < 0.05, but the range of 95% CI for OR was widely. A study with larger sample size is needed to confirm our findings. Secondly, although we excluded patients with any pre-existing heart disease based on clinical records or echocardiography, some patients might have developed subclinical heart disease after their last echocardiography. Thirdly, the determination of RV volume load may be imperfect, more accurate RV volume measurement should be performed in the future study. Fourthly, as all the participants were enrolled within 24 h of entering the ICU, the treatments prior to inclusion were implemented in other departments, the detailed information could not be obtained accurately, we will pay more attention to the collection of relevant information in future studies.

## Conclusion

In general, this study challenges traditional fluid resuscitation, which is frequently used in everyday practice. We found negative fluid balance will not always lead to a decrease, even an increase, in LVSV in patients with high CVP (≥8 mmHg) especially combined with RV_D_/LV_D_ ratio ≥0.6. The underling mechanism may be related to the filling state of RV. High CVP value and RV_D_/LV_D_ ratio ≥0.6 were significantly associated with RV stressed. Further studies of whether precise fluid management can improve patients' 28-day mortality, shorten ICU stay, or shorten the duration of mechanical ventilation are required.

## Data Availability Statement

The raw data supporting the conclusions of this article will be made available by the authors, without undue reservation.

## Ethics Statement

The research protocol was reviewed and approved by Ethics Committee of Peking Union Medical College Hospital (PUMCH-S617). The patients/participants provided their written informed consent to participate in this study.

## Author Contributions

LD and WX: conception and design, review, and revision of the manuscript. DX and ZH: data extraction. ZH: statistical analysis, interpretation of data, and writing. All authors contributed to the article and approved the submitted version.

## Conflict of Interest

The authors declare that the research was conducted in the absence of any commercial or financial relationships that could be construed as a potential conflict of interest.

## Publisher's Note

All claims expressed in this article are solely those of the authors and do not necessarily represent those of their affiliated organizations, or those of the publisher, the editors and the reviewers. Any product that may be evaluated in this article, or claim that may be made by its manufacturer, is not guaranteed or endorsed by the publisher.
