# Supplementary material for: High Central Venous Pressure and Right Ventricle Size Are Related to Non-decreased Left Ventricle Stroke Volume After Negative Fluid Balance in Critically Ill Patients: A Single Prospective Observational Study
Source: Front Med (Lausanne). 2021 Aug 31;8:715099. doi: 10.3389/fmed.2021.715099 (PMC8438320; doi:10.3389/fmed.2021.715099)
Supplement: Supplementary file 1 [file Data_Sheet_1.pdf]

Echocardiography measurement:

The five views included :parasternal long axis, parasternal short axis, apical four-chamber, subcostal four-chamber and subcostal inferior vena cava (IVC) view. Among all variables prospectively recorded in the cohort, The following parameters were especially analyzed: RV size, tricuspid annular plane systolic excursion (TAPSE), diameter of the inferior vena cava (DIVC), left ventricular eject fraction (LVEF), and left ventricular stroke volume (LVSV). LVSV was calculated by combining the averaged left ventricular outflow tract velocity time integral (LVOT VTI) by pulsed wave Doppler for the whole respiratory cycle with 2D measurement of the related diameter [16]. RV size was also evaluated at end-expiration by the RV_D/LV_D ratio. RV_D and LV_D were measured in the apical 4-CH view by identifying the maximal distance between the ventricular endocardium and the interventricular septum perpendicular to the long axis at the beginning of the QRS complex [17]， TAPSE was measured at end-expiration with the M-mode study in an apical 4-chamber view as recommended . DIVC was measured at end-expiration with the M-mode study in an subcostal inferior vena cava (IVC) view as recommended.

Hemodymic parameters measurement:

CVP measurement： Using an indwelling central venous catheter via the internal jugular or subclavian vein, a pressure sensor was connected to a monitor (Philips). While the patients were supine, the sensor was positioned in the axillary line at the fourth intercostal level. The pressure waveform and CVP values were read at the end expiratory and recorded by ICU physicians. Central venous oxygen saturation (ScVO2), central venous-arterial carbon dioxide difference (P(v-a)CO2), and serum lactate levels

(lac) : Arterial blood and superior vena cava blood were collected simultaneously. Arterial and venous blood samples were tested by a blood gas analyzer (GEMPREMIER™ 3000). $ScvO_2$ and lactate were read directly from the results. $P_{(v-a)}CO_2$ was calculated by $PvCO_2$- $PaCO_2$.
